# Paradoxical Regulation of α7nAChR and NLRP3 Inflammasome in Gastrointestinal Cancers and Ulcerative Colitis

**DOI:** 10.3390/metabo15090622

**Published:** 2025-09-18

**Authors:** Gulten Ates, Ilker Ozgur, Ismail Cem Sormaz

**Affiliations:** 1 Department of Physiology, Faculty of Medicine, Istanbul Yeni Yuzyil University, Istanbul 34010, Turkey; gultenates.ulucay@yeniyuzyil.edu.tr; 2 Department of General Surgery, Istanbul Medical Faculty, Istanbul University, Istanbul 34390, Turkey; ismail.sormaz@istanbul.edu.tr

**Keywords:** GI cancer, α7nAChR, NLRP3, inflammasome, cholinergic anti-inflammatory pathway

## Abstract

**Background:** Gastrointestinal (GI) cancers are common and pose a major public health issue. An inflammatory microenvironment drives their development and progression. The α7nAChR receptor, known to suppress autoimmune and inflammatory bowel diseases, is also linked to colorectal cancer. It enhances anti-inflammatory activity, influences tumor growth, metastasis, and treatment response, and is associated with tobacco use. NLRP3, a key inflammatory mediator, connects immunity and cancer. The α7nAChR receptor modulates tumorigenesis and therapy response by suppressing inflammatory pathways, while also regulating NLRP3 inflammasome activation through inhibition of mitochondrial DNA release. This study examines α7nAChR and NLRP3 expression in gastric and colorectal cancers, colitis, and normal tissues to clarify pathogenic mechanisms and identify therapeutic targets. **Methods:** Tissue samples of gastric tumor (S-Tm) (n = 10), colorectal tumor (C-Tm) (n = 10), colitis (UC) (n = 10), healthy stomach (S-C) (n = 10) and healthy colorectal tissue (C-C) (n = 10) taken during routine endoscopy protocols were homogenized. The α7nAChR and NLRP3 levels were examined using the ELISA method, and groups were compared. **Results:** We identified statistically significant differences in α7nAChR levels between the S-C and S-Tm (*p* < 0.05), C-C and C-Tm (*p* < 0.05), and S-C and C-Tm (*p* < 0.001) groups. The NRLP3 levels also differed significantly between the UC and C-Tm (*p* < 0.05), the S-C and C-Tm (*p* < 0.01), and the C-C and C-Tm groups (*p* < 0.01). **Conclusions:** Paradoxically, given the inflammatory regulatory role and oncogenic effects of α7nAChR, the relationship between α7nAChR and NLRP3 has become an important target for both oncological and inflammatory therapeutic approaches, particularly in inflammation-related GI cancers.

## 1. Introduction

Gastrointestinal (GI) tract cancer is a common term that includes different types of cancers that usually affect the digestive system. These cancers originate from cells in the esophagus, stomach, exocrine pancreas, liver, gallbladder, bile duct, small intestine, colon, rectum, and anus [1]. They are among the most common types of malignancies and represent a significant public health problem with a significant burden on patients and societies worldwide. Remarkably, colorectal cancer and gastric cancer are the leading causes of cancer-related deaths, accounting for approximately 25% of all cancer-related deaths [2]. GI cancers are classified as multifactorial diseases that arise from many different causes, including chronic inflammation and infection, environmental carcinogenic risk factors, and genetic predisposition in individuals [3]. In recent years, experimental studies in humans and animals have suggested that nicotinic acetylcholine (ACh) receptors (nAChRs) have important effects on the initiation, progression, and metastasis of various cancers [4]. Among the different subtypes of nAChRs, the homo-pentameric alpha 7 subtype of nAChR (α7nAChR) has a particular importance in cancer research and has been shown to be one of the main regulators in various types of cancer [5]. These receptors are named according to their activation by ACh, the dominant endogenous ligand, and nicotine, an exogenous substance.

It has been reported that the ⍺-7 nicotinic acetylcholine receptor plays a role in the cholinergic anti-inflammatory pathway and suppresses pro-inflammatory cytokine levels and increases anti-inflammatory cytokine levels, thus providing an anti-inflammatory response and immunomodulation [6]. However, deterioration, irregularity, an increase or decrease in the activity of these receptors can disrupt immunomodulation and immune tolerance in many systems, such as the gastrointestinal system, leading to autoimmune diseases, or their overexpression can cause GI cancer. In a study, it was shown that upregulation of nicotinic receptors reduces inflammation in Crohn’s disease. However, it has been reported that overexpression of nicotinic receptors can cause various types of cancer, such as colon, breast, and lung cancer, and it has been suggested that it may be one of the mechanisms that play a role in the relationship between smoking and cancer [7]. It has also been reported that nicotine acts as an autocrine and paracrine growth factor in colon cancer cells via ⍺7nAChR [8]. In addition, it has been reported that the secreted Ly-6/uPAR-related protein-1 (SLURP-1), an endogenous peptide antagonist of α7nAChR, has an anti-proliferative effect and significantly suppresses the development and progression of cancer-related features in various cancer cell lines [6].

Inflammasomes have emerged in recent years and are defined as vital players in innate immunity. Among inflammatory mediators, nucleotide-binding oligomerization domain (NOD)-like receptor protein (NLRP)-3 is one of the most popular forms today. It is considered to be the link between inflammation and autoimmunity [9,10]. Studies have shown that increased α7nAChR expression inhibits the NLRP3 inflammasome-associated inflammatory response [11,12].

Our study aimed to examine the levels of ⍺7nAChR for the cholinergic activity and NLRP3 inflammasome in healthy individuals, individuals with GI tumors (gastric and colorectal cancers), and individuals diagnosed with colitis, and to elucidate the correlation between these parameters and diseases.

## 2. Materials and Methods

### 2.1. Experimental Design

This study was approved by the Istanbul University, Istanbul Faculty of Medicine Clinical Research Ethics Committee (01.11.2024/1913). Following a comprehensive explanation of all procedures applied within the scope of the study, the participants were invited to sign an “Informed Consent” form, thereby indicating that their consent had been obtained.

### 2.2. Definition of Study Groups

In our study, diseased tissues from individuals diagnosed with GI tumors or colitis and healthy tissues from patients without known colitis or GI tumors were collected for the purpose of examining ⍺7nAChR and NLRPP-3 inflammasome levels. All participants were between 18 and 65 years old. Those with any chronic disease, cancer patients whose primary cancer was not of the stomach or colon, and those receiving neoadjuvant therapy were excluded from the study. Patients self-reported their smoking amounts in packs per year.

### 2.3. Study Groups

During routine screenings, samples were collected from patients over the age of 18 who underwent endoscopy and had no known chronic diseases:

1. Healthy Control-Stomach (S-C) (n = 10): Healthy stomach tissue without inflammatory or tumorous pathology;
2. Healthy Control-Colorectal Tissue (C-C) (n = 10): Colorectal tissue samples without inflammatory or tumorous pathology;
3. Gastric Cancer (S-Tm) (n = 10): Tumor-containing stomach tissue from patients diagnosed with stomach cancer;
4. Colorectal Cancer (C-Tm) (n = 10): Tumor-containing colorectal tissue from patients diagnosed with colorectal cancer;
5. Colitis (UC) (n = 10): Colorectal tissue with inflammatory changes from patients diagnosed with colitis.

### 2.4. Endoscopy and Colonoscopy and Sample Collection

We collected samples for the study from patients undergoing routine endoscopy, whose biopsy material revealed normal tissue, gastric cancer, colorectal cancer or colitis on pathological examination. Tissue specimens were collected during endoscopy and colonoscopy from healthy mucosa for control patients and from tumor tissue for cancer patients during macroscopic evaluation. The obtained tissue samples were washed with physiological saline, frozen in liquid nitrogen, and stored at −80 °C until the end of the study for 2 months.

### 2.5. Tissue Homogenization

For Enzyme-Linked Immuno Sorbent Assay (ELISA) kit studies, 0.15 M KCl solution was added to the weighed tissue samples, which were then homogenized using a Janke and Kunkel tissue homogenizer to prepare 10% homogenates. After centrifugation at 3000 rpm for 5 min, the supernatants were separated, and the study was conducted using this phase.

### 2.6. ELISA Method

From stomach and colorectal tissue homogenates, α7nAChR (Cat: ELK4287; Lot: 14020557, ELK Biotechnology^®^, Sugar Land, TX, USA) and NLRP3 (Cat: ELK5399; ELK Biotechnology^®^, Sugar Land, TX, USA) protein levels were measured using antibody-coated 96-well ELISA kits specific for each protein. The assays employed a biotin-based double antibody sandwich method. The same person performed tissue sampling, and the tumor location from which the samples were taken was standardized for similar patient populations. Samples were processed blindly to avoid bias, and kit standardization was performed. Samples were also backed up.

### 2.7. Statistical Analysis of Data

The statistical evaluation of the obtained results was performed using the IBM SPSS Statistics 25.0 (SPSS Inc., IBM, Chicago, IL, USA) program. The normality of the data distribution was assessed using the Shapiro–Wilk test. While evaluating the study data, descriptive statistical methods (mean, standard deviation, frequency) were used, and for quantitative data showing a normal distribution, One-Way Analysis of Variance (ANOVA) was used for comparisons between more than two groups, while the Kruskal–Wallis test was used for those not showing a normal distribution. The post hoc Tukey test was used to determine the group that caused the difference. The Pearson correlation test was used for correlation analysis of normally distributed data. Data are presented as mean ± standard deviation (SD). Significance was evaluated at the *p* < 0.05 level.

## 3. Results

### 3.1. Determining the Correlation Between Healthy Individuals and Patients with Cancer and Colitis Who Smoke

Among the adult participants (18–65 years of age) participating in the study, the female/male ratio for all experimental groups was 5/5, 5/5, 6/4, 4/6, 2/8, respectively. Smoking rates were calculated as percentages and found to be 16, 46, 20, 59, and 47, respectively (Table 1).

**Figure 1:**
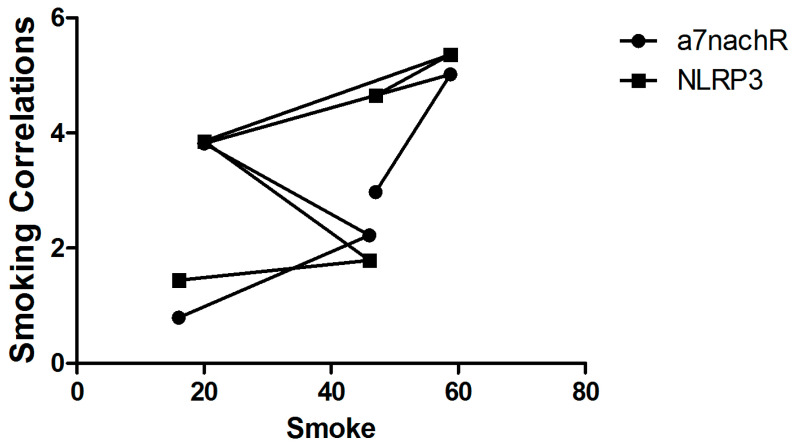
The correlations between smoking α7nAChR and NLRP3 levels (n = 10 for each group).

In our correlation analysis based on group averages, rather than individual data, no correlation was found between smoking rates and α7nAChR and NLRP3 levels (*p* > 0.05) (Figure 1).

### 3.2. Findings of α7nAChR Levels

**Figure 2:**
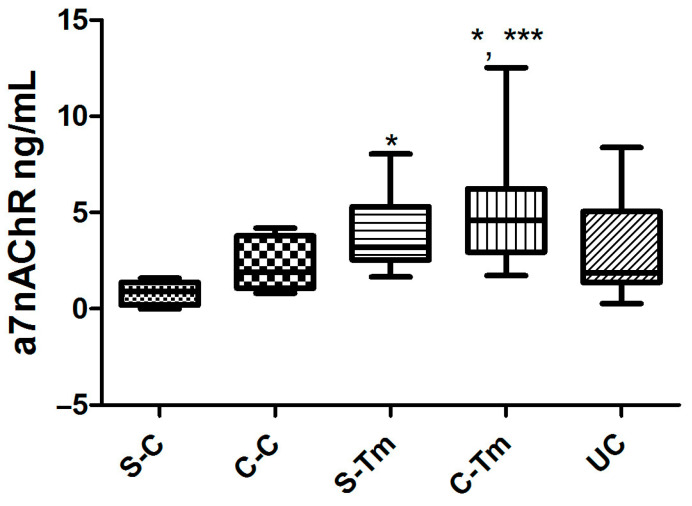
The levels of α7nAChR (ng/mL) (*: *p* < 0.05; ***: *p* < 0.001) (n = 10 for each group).

There were significant differences in α7nAChR levels [F = 6.009, *p* = 0.0006]. Post hoc comparisons using the Tukey HSD test revealed that α7nAChR levels were significantly higher in the S-Tm and C-Tm groups (*p* < 0.05) than in the S-C group (*p* < 0.001). There were also significant increases in the C-Tm group compared to the C-C group (*p* < 0.05). There were no significant differences between the other experimental groups (*p* > 0.05) (Figure 2).

### 3.3. Findings of NLRP3 Inflammasome Levels

**Figure 3:**
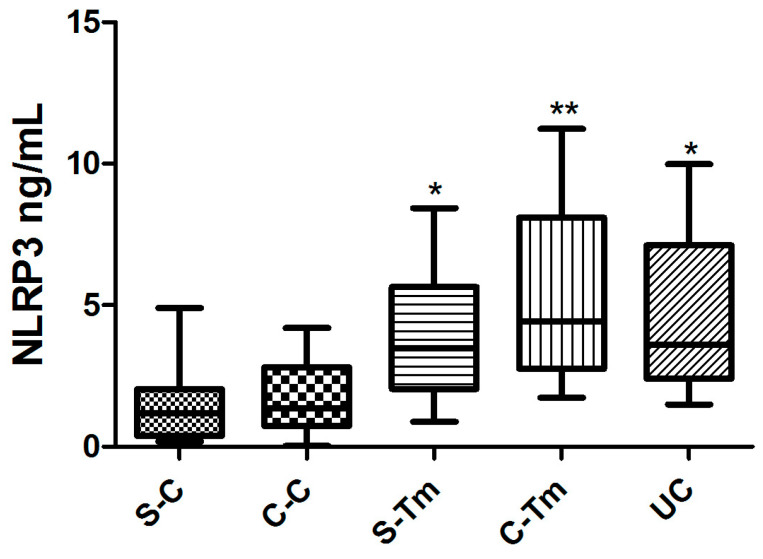
The levels of the NLRP3 (ng/mL) (*: *p* < 0.05; **: *p* < 0.01) (n = 10 for each group).

There were significant differences in levels of NLRP3 [F = 5.603, *p* = 0.0010]. In post hoc comparisons using the Tukey HSD test, statistically significant discrepancies in NRLP3 levels were identified between the UC and C-Tm groups (*p* < 0.05), as well as between the S-C and C-Tm groups (*p* < 0.01). Moreover, a substantial discrepancy in NRLP3 levels was evident between the C-C and C-Tm groups (*p* < 0.01) (Figure 3).

## 4. Discussion

Approximately two hundred years ago, Rudolf Virchow observed the presence of inflammatory cells in tumor tissues. This observation led him to hypothesize that persistent inflammation might play a pivotal role in the development of carcinogenesis. Subsequent to these findings, approximately 25% of all cancers have been identified as associated with chronic inflammation. Furthermore, mounting evidence indicates that this inflammatory condition may act as a precursor for some types of cancer [10]. GI cancers are among the most common cancers linked to chronic inflammation [13]. It has been hypothesized that the inflammatory microenvironment constitutes an intrinsic niche for the development and progression of these tumors [14]. Recent research has focused on investigating the causal relationship between the activation of inflammatory transcriptional factors, such as nuclear factor-kappaB (NF-κB), and the production of pro-inflammatory cytokines, including tumor necrosis factor-alpha (TNF-α), and their role in the development and progression of cancer [15,16]. The process of inflammation has been shown to promote the proliferation, angiogenesis, and metastasis of tumor cells. On the other hand, epidemiological studies suggest that anti-inflammatory drugs reduce the incidence and mortality rate of GI cancers. It is therefore posited that the inhibition of inflammatory transcription factors and the downregulation of pro-inflammatory cytokines may have a role in cancer treatment [17].

In their study, Tracey et al. observed that both ACh and nicotine were able to attenuate the pro-inflammatory effects of macrophages [18]. This observation gave rise to the hypothesis that the anti-inflammatory effects of ACh are mediated through peripheral nicotinic receptors. In an in vivo setting, it has been demonstrated that the transection (vagotomy) of the nerve in rats subjected to LPS-induced endotoxemia results in an augmented systemic inflammatory response [19]. This response was characterized by an earlier onset of shock and elevated serum and liver levels of the pro-inflammatory cytokine TNF-α, released from macrophages. However, electrical stimulation of the distal branch of the severed vagus was reported to attenuate this response. The present findings demonstrate that, contrary to previous suppositions, the vagus nerve can also modulate the inflammatory response by means of efferent projections of the cholinergic anti-inflammatory pathway (CAP). The term “CAP” was first coined in 2000 to denote a neural mechanism that functions to inhibit inflammation [20]. It has been demonstrated that CAP exerts its regulatory influence over the immune system by means of cholinergic mechanisms operating on ⍺7nAChR, which is expressed on macrophages and immune cells. Consequently, these receptors have been posited as a significant therapeutic target for inflammation-related cancers. The efficacy of these agents has been demonstrated, particularly in the context of diseases such as ulcerative colitis, where inflammation can lead to cancer development. On the other hand, the cancer-promoting effects of tobacco and its derivatives, and the potential for this effect to be mediated through these receptors, have raised the question of a potential drawback and dual effect.

Research has indicated that this paradoxical condition can be modified by exogenous and endogenous stimuli, and that α7nAChR should be regarded as a significant therapeutic target [21,22]. In the present study, α7nAChR levels were found to be significantly elevated in patients diagnosed with colorectal and gastric cancers in comparison to the other study groups. Conversely, these levels were found to be significantly lower in patients diagnosed with ulcerative colitis than in those diagnosed with colon cancer, and higher than in the control group.

The immune and inflammatory cells that are part of the body’s natural defenses have special receptors called Pattern Recognition Receptors (PRRs). These receptors can recognize molecules that are often found in pathogens called Pathogen-Associated Molecular Patterns (PAMPs) [23]. They can also recognize molecules released by damaged cells called Damage-Associated Molecular Patterns (DAMPs). In recent studies, the NLRP3 inflammasome has been recognized as a general sensor of cellular damage that responds to both PAMPs and DAMPs [24]. These findings highlight the importance of the NLRP3 pathway as a potential therapeutic target. Recent studies have shown that activating α7nAChR can inhibit the NLRP3 inflammasome by preventing mitochondrial DNA release in peripheral macrophages [9,10,22].

As previously mentioned, since gastrointestinal cancers are associated with inflammation, we examined NLRP3 levels in patients with gastrointestinal cancers and ulcerative colitis, an inflammatory disease involving impaired immune tolerance. We found significant differences in NLRP3 levels between gastric controls, colon tumors, ulcerative colitis patients, and healthy colons. Although an increase in inflammasomes was detected between cancerous and healthy tissue, an increase in NLRP3 levels was found in ulcerative colitis patients. However, no significant increase was found compared to the control colon group. However, no significant difference was found between colon tumors and ulcerative colitis. These results suggest that inflammasome levels are increased in GI cancers and ulcerative colitis. It has been reported that ROS generation may be involved in these mechanisms, as ROS can activate the NLRP3 inflammasome, and activated α7nAChR can inhibit oxidative stress. A recent study reported that α7nAChR activation helps control neuroinflammation in mice with autoimmune encephalomyelitis by inhibiting the NLRP3 inflammasome [11,12].

Our study is limited due to a small sample size, while still providing preliminary results and an initial framework for understanding the interplay between α7nAChR and NLRP3 in gastrointestinal cancers and ulcerative colitis. The findings establish potential correlations, but the limited sample size constrains the statistical robustness and generalizability of the results. Future studies with larger cohorts and comprehensive molecular analyses, including mechanistic and functional assays, are warranted to validate these findings. Elucidating these pathways may advance our understanding of inflammation-driven tumorigenesis and support the development of targeted therapeutic strategies for GI malignancies and inflammatory bowel diseases.

The parallel increase in α7nAChR and NLRP3 levels suggests that inflammation fosters a tumorigenic microenvironment in GI cancers, with these receptors potentially contributing to tumor migration and proliferation. Furthermore, tobacco and tobacco products exert carcinogenic effects due to the overexpression of these receptors. In our study, self-reported annual cigarette consumption in packs per day was found to be significantly higher in patients with stomach and colorectal cancer and ulcerative colitis compared to controls. Considering the relationship between cancer and smoking, this higher rate in cancer patients is consistent with the literature. However, in the ulcerative colitis group, cigarette consumption is thought to balance inflammation by stimulating CAP via nicotinic receptors. However, the literature also indicates that smoking predisposes to autoimmune diseases such as ulcerative colitis. Studies have shown that the difference between endogenous and exogenous stimulation of nicotinic receptors influences whether the receptor exerts inflammatory or anti-inflammatory effects [4]. However, as reported in other literature, α7nAChR levels may paradoxically increase in response to inflammation, perhaps to regulate it. These two conditions make the relationship between α7nAChR and NLRP3 an important target for oncological and inflammatory research. While this paradoxical effect has been mentioned in previous studies, our research is significant because no other studies have compared colitis and GI cancers with α7nAChR and its antagonist molecule.

## Figures and Tables

**Table 1:** Demographic and laboratory data of the studied groups (Mean ± SD).

Characteristics	S-C	C-C	S-Tm	C-Tm	UC
Age (Years)	47.4 ± 3.85	59.1 ± 4.2	54.2 ± 2.30	62.3 ± 3.4	49.5 ± 6.8
Sex (F/M)	5/5	6/4	5/5	4/6	2/8
Smoking (%)	16	20	46 *	58.8 *	47 *
⍺7nAChR (ng/mL) (Mean ± SD)	0.79 ± 0.60	2.22 ± 1.3 *	3.82 ± 1.9 *^,^**	5.02 ± 3.1	2.97 ± 2.5
NLPR3 (ng/mL) (Mean ± SD)	1.44 ± 1.4	1.79 ± 1.38	3.86 ± 2.36 *	5.36 ± 3.17 **	4.65 ± 2.83 *

Standard deviation (SD); F: female; M: male; α7nAChR: alpha-7 nicotinic acetylcholine receptor; NLRP3: nucleotide-binding oligomerization domain (NOD)-like receptor protein; *: *p* < 0.05; **: *p* < 0.0; (n = 10 for each group).

## Data Availability

The data presented in this study are available on request from the corresponding author.
